# Summary of the best evidence for risk stratification of exercise rehabilitation in patients with a cardiac implantable electronic device

**DOI:** 10.3389/fcvm.2024.1455486

**Published:** 2024-11-25

**Authors:** Ruiqing Di, Zheng Huang, Huifang Huang, Siyu Li, Xing Gao, Jingshuang Bai

**Affiliations:** ^1^ Nursing Department, The First Affiliated Hospital of Zhengzhou University, Zhengzhou, China; ^2^ Department of Thoracic Surgery, The First Affiliated Hospital of Zhengzhou University, Zhengzhou, China; ^3^ Department of Cardiovascular Medicine, The First Affiliated Hospital of Zhengzhou University, Zhengzhou, China

**Keywords:** cardiac implantable electronic device, exercise rehabilitation, risk stratification, evidence-based nursing, best evidence summary

## Abstract

**Background:**

Hierarchical management of sports risk is highly critical to ensure the safety of sports rehabilitation. Early identification, timely prevention and control of sports-related risk factors, and enhanced supervision and guidance can provide a basis for the formulation of sports programmes and the setting of sports monitoring levels.

**Objective:**

This study aimed to retrieve, evaluate, and integrate evidence for the stratified management of motor risk in patients with a cardiac implantable electronic device (CIED).

**Methods:**

We searched for evidence according to the “6S” model of evidence-based resources. CNKI, VIP, Wanfang Data, CBM, PubMed, Cochrane Iibrary, CINAHL, EMbase, Web of Science, BMJ Best Practice, Up To Date, and International Guidelines Collaboration Network were searched from inception to February 2024. To search for evidence on stratified management of motor risk in patients with CIEDs, this research includes guidelines, systematic reviews, meta-analyses, expert consensus, clinical decision-making, and randomized controlled trials. After methodological quality evaluation, the evidence was extracted and summarized accordingly.

**Results:**

According to the inclusion and exclusion criteria, 16 pieces of evidence were screened, including 5 guidelines, 1 clinical decision-making, 5 systematic reviews, 4 expert consensus, and 1 randomized controlled trial. After reading, extracting, and categorizing, 34 pieces of evidence in 4 areas were identified, namely, screening and assessment of exercise risk in CIEDs, exercise monitoring, implementation of exercise prescriptions, and prevention and management of exercise-related risks.

**Conclusions:**

This study provides the best evidence for the prevention and management of exercise risk in patients with CIEDs, clarifies the role of nurses in evaluating, monitoring, and educating patients undergoing motor rehabilitation, and provides a basis for the formulation of clinically feasible rehabilitation programs.

**Systematic Review Registration:**

PROSPERO, identifier (CRD2024509622).

## 1 Introduction

According to the epidemiological survey data on sudden cardiac death in China, it is estimated that there are 544,000 sudden cardiac death events in the country annually, of which more than 80% are caused by malignant arrhythmia (1–3). The prevention and treatment of arrhythmia has consistently been a research hot spot in the field of cardiovascular disease. More recently, the rapid development and application of instrument therapy technology has changed the status of cardiac arrhythmia diagnosis and treatment in China. Over the past 60 years, CIEDs have become established as an essential therapeutic modality of cardiovascular care for the treatment of patients with bradycardia, tachycardia, and heart failure (4, 5). Currently, the number of individuals with CIEDs worldwide has increased drastically, and hundreds of thousands of new patients join this cohort every year (6). CIEDs include implantable cardioverter-defibrillators (ICDs), cardiac resynchronization therapy-pacemakers/defibrillators(CRT/CRT-D), permanent pacemakers(PM) and leadless pacemakers.

CIED recipients are considered eligible for motor rehabilitation programs (7). Moderate leisure-time physical activity is safe and clinically recommended for most patients with CIEDs (8). Thus, strenuous exercise increases the risk of arrhythmias. Patients with CIED should receive special attention as their needs may differ from those of other patients participating in Cardiac rehabilitation (CR). This is not only related to the underlying heart disease but also to specific issues such as psychological adjustment to living with an implanted device. Heart failure patients with ICD are often reluctant to participate in motor rehabilitation programs due to fear of shock from exercise training (9, 10). Moreover, patients with a CIED have a higher risk of arrhythmia, syncope, and sudden cardiac death (11, 12). The occurrence of a supraventricular arrhythmia may cause an inappropriate shock to the cardioverter, so heart rate monitoring should be enhanced in advance to prevent it.

Motor rehabilitation for patients with CIED is a unique opportunity not only to optimize medical treatment, increase exercise capacity, and improve the patients’clinical condition, but also to supervise the correct functioning of the device (13, 14). Nurses should manage risk stratification for exercise rehabilitation and continuously monitor these patients during exercise training. Efforts should be made to improve patient participation in sports rehabilitation, provided patient safety is ensured.

More generally, there is still room for improvement in the assessment, monitoring and intervention of exercise risk by medical institutions. Therefore, summarizing the available evidence is essential to prevent the risks associated with CIEDs during physical activity. As evidence from studies on motor rehabilitation in patients with CIED is scattered, available evidence was summarized in the systematic integration of screening and assessment of exercise risk in CIED, exercise monitoring, implementation of exercise prescriptions, and prevention and management of exercise-related risks to provide a basis for clinical rehabilitation strategies.

## 2 Methods

### 2.1 Inclusion and exclusion criteria

The PIPOST model was used to construct an evidence summary for risk stratification management of sports rehabilitation for patients with CIEDs (15). The inclusion criteria were as follows: (1) The target population of the research application were patients with CIEDs who were ≥18 years old; (2) The intervention involved inspiratory muscle training; aerobic training; resistance training; low-intensity training; moderate intensity aerobic exercise; high-intensity interval training; cardiac rehabilitation; sports rehabilitation; activity monitoring; rehabilitation at home; and remote monitoring; (3) The professionals who applied the evidence were clinical staff; (4) The outcome measures were exercise-related risk events, such as sudden cardiac death and arrhythmia, exercise intensity, mortality, and readmission rates; (5) The place of evidence was the hospital; and (6) Research types include best practice manuals, clinical decisions, guidelines, expert consensus, evidence summaries, systematic reviews, and RCT. The exclusion criteria were as follows: (1) Document type: abstract, proposal, draft, report; compendium of conference papers, incomplete information or non-availability of full texts; (2) Systematic reviews or meta-analyses that have been adopted by the guidelines; and (3) Guideline interpretation or guideline application effect evaluation.

### 2.2 Search strategy and data sources

The evidence was searched according to the “6S” model of evidence-based resources (16). CNKI, VIP, Wanfang Data, CBM, PubMed, Cochrane Iibrary, CINAHL, EMbase, Web of Science, BMJ Best Practice, Up To Date, National Institute for Health and Care Excellence (NICE), JBI Centre for Evidence-based Health Care Database, International Guidelines Collaboration Network, National Guidelines Library of the United States, Canadian Medical Association Clinical Practice Guidelines Library, and Scottish Intercollegiate Collaboration Network were searched from inception to February 2024. The English search terms were as follows: “cardiac implantable electronic devices/pacemaker, artificial/defibrillators, implantable/Cardiac Resynchronization Therapy/Cardiac Resynchronization Therapy Devices,” “exercise training/risk evaluation/risk stratification/wearable monitor/remote monitoring/exercise-based cardiac rehabilitation/low intensity training/resistance training/aerobic training/moderate continuous training/high-intensity interval training,” and “Sudden cardiac/arrhythmia/Readmission/mortality.” The language was either Chinese or English. Using the English search strategy in PubMed provided the following outputs in Table 1.

**Table 1:** Literature search strategies are derived from pubmed database.

Search	Query	Items found
#1	[cardiac implantable electronic devices(Title/Abstract)] OR [pacemaker, artificial(MeSH Terms)]) OR [defibrillators, implantable(MeSH Terms)]) OR [Cardiac Resynchronization Therapy(MeSH Terms)]) OR [Cardiac Resynchronization Therapy Devices(MeSH Terms)]	49,854
#2	[exercise training(Title/Abstract)]) OR [risk factor*(Title/Abstract)]) OR [risk assess*(Title/Abstract)]) OR [risk evaluat*(Title/Abstract)]) OR [risk scree*(Title/Abstract)]) OR [risk predict*(Title/Abstract)]) OR [prevent(Title/Abstract)]) OR [Risk stratification(Title/Abstract)]) OR [wearable monitor(Title/Abstract)]) OR [exercise based cardiac rehabilitation(Title/Abstract)]) OR [low intensity training(Title/Abstract)]) OR [resistance training(Title/Abstract)]) OR [aerobic training(Title/Abstract)]) OR [moderate continuous training(Title/Abstract)]) OR [muscle training(Title/Abstract)]) OR [high-intensity interval training(Title/Abstract)]	1,519,062
#3	[Readmission(Title/Abstract)] OR [mortality(Title/Abstract)]) OR [Sudden cardiac(Title/Abstract)]) OR [arrhythmia(Title/Abstract)]) OR [arm disability(Title/Abstract)]) OR [Upper limb dysfunction(Title/Abstract)]) OR [Shoulder range of motion(Title/Abstract)]	1,122,736
#4	[systematic review(Title/Abstract)] OR [meta-analysis(Title/Abstract)]) OR [evidence summar*(Title/Abstract)]) OR [guideline*(Title/Abstract)]) OR [evidence based nursing(Title/Abstract)]) OR [consensus(Title/Abstract)]) OR [clinical practice(Title/Abstract)]) OR [best practice*(Title/Abstract)]) OR [Randomiz* (Title/Abstract)]) OR [review, systematic(MeSH Terms)]) OR [clinical practice guideline(MeSH Terms)]) OR [clinical trials, randomized(MeSH Terms)]	1,972,029
	#1 and #2 and #3 and #4	504

### 2.3 Literature quality evaluation

AGREE II (17) was applied for clinical guideline study assessment. The literature obtained was evaluated by four independent reviewers. The tool includes 23 items in six areas, including the scope and purpose, participants, rigor of the formulation, clarity of presentation, applicability and editorial independence. Each item was scored on a scale of 1 to 7 points (1 point = “strongly disagree,” 7 point = “strongly agree”), and the standardized percentage was used as the final score for each area.

$$\begin{array}{rl} & \mathrm{Standardized}\;\mathrm{percentage}=\\ & (\mathrm{actual}\;\mathrm{score}\;-\;\mathrm{lowest}\;\mathrm{possible}\;\mathrm{score})/\\ & (\mathrm{highest}\;\mathrm{possible}\;\mathrm{score}\;-\;\mathrm{lowest}\;\mathrm{possible}\;\mathrm{score})\;\times 100\text{\%}. \end{array}$$

The standardized percentage score in all fields was used to classify the evaluation grades as follows: Grade A (recommended): All fields scored ≥60%; Grade B (recommended after modification and improvement): the number of fields scored ≥30% was ≥3, but there were fields with scores <60%; and C level (not recommended): the number of fields scored <30% was ≥3.

The expert consensus was evaluated using the JBI Evidence-Based Health Care Center evaluation standard (2017 edition) (18). The evaluation standard contains of six items, which were evaluated as “yes,” “no,” “unclear,” or “not applicable.” The systematic reviews were evaluated using the JBI Evidence-Based Health care Center System Evaluation Standard (2016 edition) (19). The tool included 11 evaluation items, including evidence-based problem definition, search strategy, literature quality evaluation, etc. Each item was evaluated as “yes,” “no,” “unclear,” or “not applicable.” Retrospective reference methods were used to evaluate the methodological quality of the studies recommended for inclusion in clinical decisions.

The methodological quality of the randomized controlled trials (RCTs) was evaluated using the JBI Evidence-Based Health Care Center Evaluation Tool (2016) (20), which included 13 evaluation items. The evaluators were required to make a judgment of “yes,” “no,” “unclear,” or “not applicable” for each evaluation item. The included literature consisted of at least two authors. The researchers were systematically trained in evidence-based methodologies and independently completed evaluations. In cases of conflicts of opinion, the group discussed the discrepancies. A third researcher on the team participated in these discussions to ensure that a final common conclusion was reached.

### 2.4 Summary and classification of evidence

The evidence production team consisted of two evidence-based nursing experts and two nursing graduate students who had been trained by the evidence-based education system. All members of the team have clinical experience and evidence-based practice experience in cardiovascular disease nursing. The researchers read the included literature and extracted evidence related to the topic, including the evidence items, the content of evidence, sources of evidence, types of included literature, the authors, and the year of publication. Regarding evidence from clinical guidelines and the evidence summary, the original grading system was adopted. Regarding evidence from other sources, due to the lack of a corresponding grading system, the JBI evidence pre-grading and evidence recommendation level system (2014 edition) was used to grade the evidence according to the types of studies that included the evidence in the original literature (21). In the process of evidence integration, when the conclusions of different authors are found to be conflicting, the principle of high-quality evidence priority and the latest published evidence priority is followed. The classification of evidence was independently completed by two researchers. If there was any disagreement, a third researcher in the team was invited to participate in the discussion. If necessary, an expert meeting was arranged until a consensus was reached.

## 3 Results

### 3.1 Literature search results

**Figure 1:**
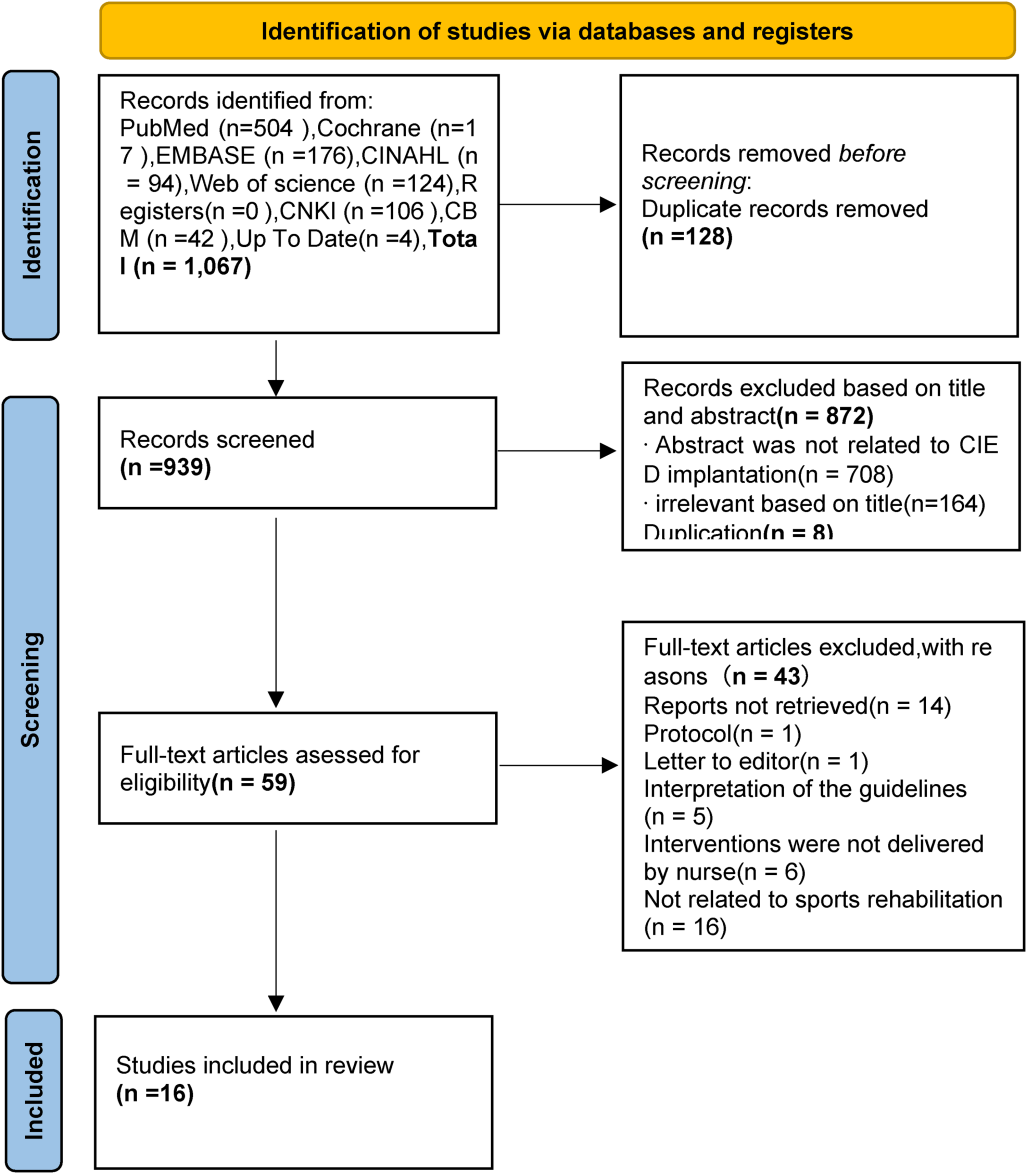
PRISMA flow diagram of literature search process.

The Preferred Reporting Items for Systematic Reviews and Meta-Analyses (PRISMA) searching process is presented in Figure 1. A total of 1067 relevant articles were identified, and 128 records were found to be duplicates using Endnote. After excluding records based on titles and abstracts (872) and duplications (*n* = 8), full texts were retrieved and downloaded. A total of 59 studies remained after the initial screening process. Each of the full texts were read to determine if the article met the inclusion and exclusion criteria. Finally, 16 articles were included in this review, comprising 1 clinical decision (22), 5 guidelines (23–27), 4 expert consensus (28–31), 4 systematic reviews (10, 14, 32, 33), 1 meta-analysis (13), and 1 randomized controlled trial (6). The basic characteristics of the included literature are summarized in Table 2.

**Table 2:** Basic characteristics of the included literature (*n* = 16).

Inclusion in the literature	Title	sources	Year	Nature of literature
Kevin F. Kwaku (22)	Cardiac implantable electronic devices: Periprocedural complications	Clinical decision-making	2023	Up to date
Wilfried Mullens (23)	Optimized implementation of cardiac resynchronization therapy: a call for action for referral and optimization of care	ESC Network	2021	Evidence-based guidelines
Michael Glikson (24)	2021 ESC Guidelines on cardiac pacing and cardiac resynchronization therapy	PubMed	2021	Evidence-based guidelines
Shigeru Makita (25)	JCS/JACR 2021 Guideline on Rehabilitation in Patients With Cardiovascular Disease	PubMed	2021	Guidelines
SIGN (26)	Scottish Intercollegiate Guidelines Network (SIGN). Cardiac rehabilitation.	SIGN Network	2017	Evidence-based guidelines
NICE (27)	Implantable cardioverter defibrillators and cardiac resynchronisation therapy for arrhythmias and heart failure	NICE Network	2014	Evidence-based guidelines
Chinese Society of Arrhythmias, Chinese Society of Pacing and Electrophysiology (28)	Chinese expert consensus on leadless pacemaker (2022)	Wanfang	2022	Expert consensus
Cardiac Rehabilitation Management Committee of Chinese Hospital Association (29)	Chinese expert consensus on graded diagnosis and treatment of cardiac rehabilitation	Wanfang	2022	Expert consensus
Cardiovascular Health and Scientific Sports Branch of China Health Culture Association (30)	Chinese Expert Consensus on Evaluation and Monitoring of Exercise Related Cardiovascular Risk	Wanfang	2022	Expert consensus
Roberto F. E. Pedretti (31)	Comprehensive multicomponent cardiac rehabilitation in cardiac implantable electronic devices recipients: a consensus document from the European Association of Preventive Cardiology (EAPC; Secondary prevention and rehabilitation section) and European Heart Rhythm Association (EHRA)	PubMed	2020	Expert consensus
Li-fang Ye (10)	Efficacy and Safety of Exercise Rehabilitation for Heart Failure Patients With Cardiac Resynchronization Therapy: A Systematic Review and Meta-Analysis	EMBASE	2020	Systematic review
Kim M. Nielsen (32)	Exercise-based cardiac rehabilitation for adult patients with an implantable cardioverter defibrillator (Review)	Conchrane	2019	Systematic review
Afnan Hamad Alswyan (33)	A Systematic Review of Exercise Training in Patients With Cardiac Implantable Devices	PubMed	2018	Systematic review
Kjetil Isaksen (14)	Exercise training and cardiac rehabilitation in patients with implantable cardioverter defibrillators:a review of current literature focusing on safety, effects of exercise training, and the psychological impact of programme participation	CINAHL	2011	Systematic review
Ambarish Pandey (13)	Safety and Efficacy of Exercise Training in Patients With an Implantable Cardioverter-Defibrillator A Meta-Analysis	PubMed	2016	Meta-analysis
Gulin Findikoglu (6)	Limitation of motion and shoulder disabilities in patients with cardiac implantable electronic devices	PubMed	2015	RCT

### 3.2 Results of quality evaluation

#### 3.2.1 Quality appraisal results of the guidelines

Four clinical practice guidelines were included in this study. The percentage of standardization in six fields of three guidelines was ≥60%, and the recommendation level of three guidelines was finally classified as level A. One guideline had a standardized percentage of 41.67% in the independence area, resulting in an overall quality recommendation of level B. The AGREE Ⅱ scores for each field are presented in Table 3.

**Table 3:** Results of quality evaluation of guidelines on CIED using AGREE-Ⅱ.

Domains	Wilfried Mullens (23)	ESC (24)	JCS (25)	SIGN (26)	NICE (27)
Domain 1: Scope and purpose	86.11%	98.61%	95.83	95.83	90.28
Domain 2: Stakeholder involvement	79.17%	94.44%	79.17	97.22	81.94
Domain 3: Rigor of development	58.33%	92.71%	65.63	97.40	64.58
Domain 4: Clarity of presentation	90.28%	95.83%	69.44	95.83	88.89
Domain 5: Applicability	77.08%	95.83%	62.50	96.88	72.92
Domain 6: Editorial independence	93.75%	97.92%	93.75	97.92	95.83
Number of fields with ≥60% (one)	5	6	6	6	6
Number of fields with ≥30% (one)	6	6	6	6	6
Overall quality	B	A	A	A	A

#### 3.2.2 Quality appraisal results of the systematic review

One meta-analysis and four systematic reviews were included in this review, and the quality evaluation results are presented in Table 4. The study by Ye et al. (2020) was evaluated as “yes” for all 11, except for entry 10, “Were recommendations for policy and/or practice supported by the reported data?”, which was evaluated as “no” (10). The study by Alswyan et al. (2018) was evaluated as yes for all entries, except for entry 7, “Whether certain measures are used to reduce errors when extracting data?”, which was evaluated as “no” (33). The study by Isaksen et al. (2012) was evaluated as yes for all entries, except for entry 4, “Was the search strategy appropriate?”, “Were the sources and resources used to search for studies adequate?”, which were evaluated as “no” (14) because only searched in the PubMed database. Entry 4 refers states that a systematic review should try all available evidence and develop a comprehensive retrieval strategy. Reference 14 only searched the PUBMed database, so item 4 was judged to be a no. However, this systematic review (14) comprising 1,889 patients which with a large sample size and relatively credible results, so it was still included in this summary of evidence.

**Table 4:** Quality evaluation results of the systematic review (*n* = 5).

Included literature	(1)	(2)	(3)	(4)	(5)	(6)	(7)	(8)	(9)	(10)	(11)
Kjetil Isaksen (14)	Y	Y	Y	N	Y	Y	Y	Y	Y	Y	Y
Ambarish Pandey (13)	Y	Y	Y	Y	Y	Y	Y	Y	Y	Y	Y
Li-fang Ye (10)	Y	Y	Y	Y	Y	Y	Y	Y	Y	NA	Y
Nielsen KM (32)	Y	Y	Y	Y	Y	Y	Y	Y	Y	Y	Y
Afnan Hamad Alswya (33)	Y	Y	Y	Y	Y	Y	NA	Y	Y	Y	Y

(1) Is the review question clearly and explicitly stated? (2) Were the inclusion criteria appropriate for the review question? (3) Was the search strategy appropriate? (4) Were the sources and resources used to search for studies adequate? (5) Were the criteria for appraising studies appropriate? (6) Was critical appraisal conducted by two or more reviewers independently? (7) Whether certain measures are used to reduce errors when extracting data? (8) Were the methods used to combine studies appropriate? (9) Was the likelihood of publication bias assessed? (10) Were recommendations for policy and/or practice supported by the reported data? (11) Were the specific directives for new research appropriate? Letter Y, indicates whether criteria (1)–(6) are met and decides whether to include evidence.

#### 3.2.3 Quality appraisal results of the expert consensus

Four expert consensus were included in this study. All entries were evaluated as “yes,” and the quality evaluation results are presented in Table 5.

**Table 5:** Quality evaluation results of expert consensus (*n* = 4).

Included literature	(1)	(2)	(3)	(4)	(5)	(6)	Whether to include
Roberto F. E. Pedretti (31)	Y	Y	Y	Y	Y	Y	Y
Chinese Society of Arrhythmias, Chinese Society of Pacing and Electrophysiology (28)	Y	Y	Y	Y	Y	Y	Y
Cardiac Rehabilitation Management Committee of Chinese Hospital Association (29)	Y	Y	Y	Y	Y	Y	Y
Cardiovascular Health and Science Sports Branch of China Medical and Health Culture Association (30)	Y	Y	Y	Y	Y	Y	Y

(1) Are the sources of the ideas clearly marked? (YES/NO) (2) Does the opinion come from an influential expert in the field? (YES/NO) (3) Whether the point of view presented is centered on the relevant population interests of the study? (YES/NO) (4) Are the stated conclusions based on the analysis? Are ideas expressed logically? (YES/NO) (5) Was there any reference to other literature? (YES/NO) (6) Are there any inconsistencies between the ideas presented and the previous literature? (YES/NO). Letter Y, indicates whether criteria (1)–(6) are met and decides whether to include evidence.

#### 3.2.4 Quality appraisal results of the clinical decision

One clinical decision (22) from Up To Date was directly included. Tracing the source of this clinical decision revealed that it was an expert consensus (4), and the quality evaluation results showed that all items were evaluated as “yes.”

#### 3.2.5 Quality appraisal results of the randomized controlled trials

A randomized controlled trial (6) from PubMed was included. The evaluation results of items 5 and 6 (“Were those delivering treatment blind to treatment assignment?”, “Were outcomes assessors blind to treatment assignment?”) were evaluated as “unclear.”

### 3.3 Summary of evidence

Through evidence extraction and integration, the evidence for CIED risk stratification management precautions, with a total of 34 items of evidence, as presented in Table 6.

**Table 6:** Summary of the best evidence for risk stratification of exercise rehabilitation in patient with CIEDs.

Category of evidence	Evidence description	Source	Evidence level	Recommendation level
Screening and assessment of exercise risk in CIED	1. Evaluation of disease status: Exercise training should only be considered in stable patients receiving optimal medical treatment (13).	Meta-Analysis	2	A
	2. Clinical and technical evaluation: History and clinical examination, chest x-ray, echocardiogram, Holter monitoring, and CPET (31).	Expert consensus	5	A
	3. Guidelines recommend pre-exercise health screenings, which determine risk stratification by incorporating three core elements: exercise habits, symptoms and disease status (30).	Expert consensus	5	A
	4. Cardiovascular risk assessment in exercise populations, structural, coronary blood supply, arrhythmia, and cardiac function (30).	Expert consensus and systematic review	2	A
	5. Assess potential inducible ischaemia and arrhythmia: All patients with ICD should be assessed with an exercise stress test before entering the ET program (14).	RCT and statement	1	A
	6. Evaluation of indications of rehabilitation activities: (1) There was no current or recurrent chest pain in the past 8 h, and there was no current obvious arrhythmia: (2) The levels of creatine kinase and troponin were not elevated, and no new manifestations of heart failure decompensation appeared; (3) Resting heart rate 50–120 beats/min, resting SBP 90–150 mmHg, resting DBP 60–100 mmHg; (4) Oxygen saturation >90% or recently decreased by <4% (29).	Expert consensus	5	B
	7. Risk stratification of sports rehabilitation: (1) Low risk: EF ≥50% and METs ≥7; (2) Medium risk: EF: 40%–50% or METs: 5.0–7.0; (3) High risk: EF <40% or METs ≤5.0 or anxiety or depression (29).	Expert consensus	5	A
Exercise monitoring	8. After an initial evaluation that includes a stress test, ICD patients may be enrolled in an exercise training program under the supervision of an experienced staff member with the necessary skills in cardiopulmonary resuscitation (14).	RCT and statement	1	A
	9. Medical institutions and places associated with sports training shall be equipped with various monitoring equipment, protective measures, emergency vehicles, defibrillators, respiratory Aids (30).	Expert consensus	5	A
	10. For the general population and low-risk cardiovascular patients, in addition to self-perceived fatigue scores, a wristwatch and heart rate band can help achieve bullseye rate control. Wearable devices can also be used for sports training and risk monitoring (30).	3 systematic reviews	1	A
	11. Patients with moderate and high risk cardiovascular diseases should undergo exercise rehabilitation training under medical supervision to ensure the safety of exercise training (13, 29).	Prospective parallel arm trials	2	A
	12. Patients should also have a 12-lead electrocardiogram recorded after implantation. It is helpful to validate biventricular capture in CRT treated patients (32).	26 relevant randomised controlled trials and Expert consensus	1	A
Implementation of exercise prescriptions	13. Exercise start time: (1) Patients with leadless pacemaker can normally move down to the ground 5–6 h after surgery, and the presence of hematoma at the puncture site should be observed before moving to the ground to determine whether there is an arteriovenous fistula (22). (2) The interval time between CRT implantation and exercise training in the included studies reported was at least 2 weeks (25). (3) Waiting 6 months following ICD implantation to give time for proper standard adjustments of ICD functioning before recommending exercise (14).	2 systematic reviews and Expert consensus	1	B
	14. The CIED exercise prescription consists of endurance training, aerobic continuous training, and resistance training (31).	Expert consensus	5	A
	15. Endurance training may use a continuous and/or interval or intermittent training model, 3–5 days/week, over a 30–60 min period, associated with dynamic exercises (31).	Expert consensus	5	A
	16. The prescription for CRT continuous aerobic training may be similar to that used in HF patients, keeping in mind the device's upper limit. Aerobic exercise of up to 60 min using various modalities 2–3 times per week at 60%–80% max HR based on pre-exercise tests (14, 31).	systematic reviews	1	A
	17. Inspirational exercise can be particularly valuable in the most fragile and recently stabilized patients (31).	systematic reviews	1	A
	18. Upper body strength training may dislodge the newly implanted leads, so resistance training is not recommended during the first 4–6 weeks post-implantation. (20, 21). Resistance training should be low to moderate intensity and completed twice a week, deeply tailored to each individual patient (33).	Guidelines and Expert consensus	1	A
	19. High-intensity interval training may be considered for low-risk, stable patients (25).	Guidelines	2	A
	20. Low-intensity endurance training may be considered during the initial phase of the exercise program, or for patients with reduced exercise capacity (25).	Guidelines	2	A
	21. Exercise interventions tested to date in the CIED population have shown that moderate to high intensity exercise training is safe and effective in improving cardiopulmonary outcomes without adverse events (33).	systematic reviews	1	A
	22. The majority of studies used training at moderate to high levels of intensity, at 60%–90% of maximum HR attained during the CPET or based on age-adjusted estimated maximum HR (12 OR 14) (33).	systematic reviews	1	B
	23. It showed evidence in favor of exercise-based rehabilitation in the ICD: 8–26 weeks of aerobic training with motion frequency: 2–7 days per week and duration: 10–60 min per session (32).	systematic reviews	1	B
	24. The target heart rate during exercise training must be tailored to fall well below the ICD threshold for tachycardia detection (13).	Prospective parallel arm trials	2	A
Prevention and management of exercise-related risks	25. Exercise-related cardiovascular high-risk status was assessed if there was at least one core variable or two or more non-core variables. Core variables: (1) Age (male over 50 years, female over 60 years); (2) Combined with clear cardiovascular disease, type 2 diabetes, or kidney disease; (3) Have early onset (male less than 55 years old, female less than 65 years old) coronary heart disease or other congenital; (4) Family history of inherited heart disease; (5) Participate in or plan to participate in high-risk extreme sports (30).	Expert consensus	5	A
	26. The general risks of exercise training are falls and fractures. In particular, older and obese patients, who typically have orthopedic conditions of the lumbar spine and lower extremities, should be aware of the risk of exacerbation through exercise (25).	RCTs	1	A
	27. Continuous monitoring of CIED patients, stopping training when heart rate falls below a set ICD treatment range of 10–20 beats/min, use of beta-blockers when clinically indicated for the patient's disease, and monitoring their effect on maximal heart rate (31).	Expert consensus	5	A
	28. Documented and evaluated and approved by a physician before resuming activities, including abnormal changes in blood pressure (SBP decrease ≥10 mmHg or increase >40 mmHg), severe ventricular or atrial arrhythmias, second or third-degree atrioventricular block, signs and symptoms of intolerance to exercise (angina, significant shortness of breath, dizziness), and ECG ischemic changes (6).	Expert consensus	5	B
	29. Risk factors for motor limitation: Pain, association of CIED with pectoral muscle, a possible subtle ongoing capillary pathology, and avoidance behavior of the patient to minimize the risk of lead dislocation (6).	RCT	1	A
	30. For all patients with CIEDs, physical activities associated with a risk of chest trauma (e.g., rugby, boxing, martial arts) should be avoided (24, 31).	Expert consensus and Guideline	5	A
	31. In-hospital/discharge, shoulder joint restriction is lifted, but swimming and abduction above 90° should be avoided as much as possible until the outpatient checkup at 1 month, with re-evaluation after confirmation (25).	Guideline	1	A
	32. Living with CRT, discussion of survival consequences, treatment, lifestyle, exercise. Information through health care providers (e.g., CRT specialist, heart failure nurse) and paper and web-based education (e.g., www.heartfailurematters.org) might improve patients’ understanding and engagement (23).	Prospective parallel arm trials	2	A
	33. Psychoeducation (goal setting, self-monitoring) should be considered for patients in cardiac rehabilitation to facilitate adherence to physical activity (26).	Guideline	1	A
	34. Psycho-educational interventions, such as behavioral change models, had a positive effect on physical activity levels over 6–12 months compared to exercise and risk factor education (26).	Guideline	1	A

## 4 Discussion

### 4.1 Strengthening sports risk screening, assessment and monitoring to ensure sports safety

Evidence 1–7 summarizes the content of exercise risk screening assessment, which includes clinical and technical evaluation, pre-exercise health screening, cardiovascular risk assessment, assessment of potential inducible ischemia and arrhythmia, evaluation of the indications of rehabilitation activities, and risk stratification of motor rehabilitation. In recent years, pacemaker post-implantation rehabilitation programs have demonstrated clinical efficacy in terms of enabling patients to achieve improved functional capacity, reduced morbidity through personalized, and supervised training protocols (14, 34, 35). The importance of functional assessment through motor testing prior to the start of a training programme has been highlighted by the statement (36), and incremental cardiopulmonary exercise testing, when available, is recommended as the gold standard for physiologically integrated exercise intensity assessment and prescription (30). By performing exercise load tests on patients fitted with pacemakers, it is possible to assess not only exercise capacity and determine exercise intensity, but also the heart rate response of the pacemaker and the appropriateness of the pacemaker setting.

Evidence 8–12 summarize different exercise risk monitoring methods for low and intermediate to high-risk patients. It is recommended to monitor heart rhythm and heart rate during CIED exercise rehabilitation (31). After introducing exercise training programs, the heart rate response should be assessed through Electrocardiogram (ECG) monitoring during each exercise session (32), also note if an increase in heart rate triggers a worsening of myocardial ischemia or heart failure. In order to prevent inappropriate shocks, the following measures are recommended: exercise testing and training should be stopped at 10–20 beats/min below the programmed zones of therapies, these patients should be continuously monitored during exercise training (25), proper beta blockers should be administered, and their effect on maximum heart rate should be examined before starting a rehabilitation program. With the development of technology, it is recommended to introduce digital health tools for remote monitoring of CIED exercise rehabilitation, through the real-time data provided by the tools, to help healthcare professionals assess the condition of patients and exercise risks.

### 4.2 Exercise prescriptions should be tailored to the intensity of the exercise, following a step-by-step principle

Risk stratification management in sports rehabilitation is summarized by evidence 13–23; however, the strength of the motion and the motion start time are disputed.Cardiac events that may develop during exercise training include hypotension, arrhythmias, and exacerbations of heart failure (37). Moderate to high intensity exercise is recommended for patients with CIED, as demonstrated by evidence 21. Exercise intensity was directly related to both the amount of improvement in exercise capacity and the risk of adverse events during exercise. Successive increases in intensity during the program may induce beneficial effects such as collateral formation and improved endothelial function, which reduce ischemia during bouts of exercise. Study Group of Sports Cardiology of the Working Group of Cardiac Rehabilitation suggests that sports activity in stable patients should be at a low static or dynamic intensity (14). During the initial phase of the exercise program, or for patients with reduced exercise capacity, low-intensity endurance training may be considered. High-intensity interval training may also be considered for low-risk, stable patients (38). Although most patients undergo re-vascularization before entering an exercise training program, ischemia may be apparent in parts of the myocardium during training at high intensity in patients with coronary artery disease (14). There are various ways to determine the intensity of exercise. Achieving 80% of the patient's heart rate reserve is a sufficient target that can be assessed using the 6-minute walk test (39).

Although a number of systematic reviews are of reasonable or good quality, there is still insufficient evidence to draw firm conclusions about the onset of motion. Some reports suggest that before recommending exercise, a waiting period of 6 months following ICD implantation should be observed to allow for proper standard adjustments of ICD functioning (14). However, Kjetil Isaksen (40) suggests that exercise training can be started 2 h after ICD implantation. In cases where the content of the available evidence is conflicting, the principles of evidence-based evidence priority, high-quality evidence priority, and the priority of the latest published authoritative literature are recommended. Thus, this evidence summarizes the recommendation to initiate motor training 6 months after ICD placement. For patients with pacemakers who have normal coagulation function and excellent nutritional status, the shoulder joint can be correctly fixed and postoperative bed activity can be carried out 3–6 h after surgery (39). If bed rest is prolonged during this phase, motor capacity will decline and frailty will progress (28). Therefore, an early mobilization programme should be initiated from the bedside, in parallel with primary treatment, leading to early exercise training. In clinical practice, medical personnel can give priority to encouraging patients to “move” while ensuring their safety. The goal is to improve exercise self-efficacy by first completing exercise frequency targets and then working towards achieving standards of exercise intensity and time. Due to the differences in individual disease conditions and exercise capacity, exercise training programs should be based on a step-by-step principle based on the evaluation of benefits and risks (41). This consists of gradually increasing the movement time from minor to greater, the movement intensity from low to high, and the movement frequency from sparse to complex. Therefore, a combination of comprehensive clinical assessment and exercise-related risk assessment is recommended to develop individualized exercise rehabilitation strategies that enable patients to exercise in a deliberate, planned and scientifically appropriate manner.

### 4.3 Strengthen psychological and risk factor education with nurses in the lead role

Guidelines (25) state that cardiac rehabilitation requires the involvement of multiple specialties (cardiologists, physiotherapists, and psychologists). It is critical to give patients with CIEDs the opportunity to live an active lifestyle that spans from daily physical activities of life to exercise training. Recognize the role of nurses as health instructors in cardiac rehabilitation: Full-time nurses dedicated to “cardiovascular rehabilitation” provide rehabilitation sessions to patients with cardiovascular disease and other patients when not involved in cardiovascular rehabilitation. Nurses also provide rehabilitation knowledge to heart failure patients with implanted ICDs, CRTs or permanent pacemakers. Exercise training improves aerobic capacity in patients with ICD, its effects on anxiety, depression and quality of life are still debated. In the HF-ACTION study, exercise training in patients with an ICD seems to be safe and is not associated with an increased risk of shocks (42). Several studies have described an ICD recipient's fear of shocks due to high pulse rates, which may subsequently lead to a persistent reduction in physical activity levels. It is suggested to evaluate the patients’ stress perception, and give them stress reduction training and acceptance commitment therapy exercises to improve their fears.In risk stratification, factors such as patient psychological stress, social support systems, quality of life, and how they affect the effectiveness and safety of exercise rehabilitation can be considered. Risks associated with exercise and prevention measures are summarized in evidence 24–35. Prevention of sports injuries includes measures such as the use of appropriate training environments, proper training time, adequate preparation activities, reasonable training methods, proper exercise, and post-exercise relaxation. In order to prevent accidents during exercise training, sufficient attention should be paid not only during but also after exercise. It is also recommended that patients engage in preparatory exercises, such as stretching and thorough warm-up, at the beginning of the exercise session. At the conclusion of exercise sessions, it is advisable for patients to engage in a “cool down” period by either by running or walking at a reduced intensity and speed, or by performing consolidation exercises such as stretching. This gradual decrease in intensity helps to return the patient to their resting blood pressure and heart rate to prevent hypotension and dizziness after exercise (25). Extracting and sharing information from each team member about cardiovascular events and any complications that may occur to each patient during exercise training is critical to helping nurses explain these risks to patients in advance and take steps to minimize them while continuing to prepare for the unexpected.

## 5 Limitations

This study has several limitations. First, This article summarizes the evidence on the stratification of exercise risk in patients with CIED, but individualized exercise programs, such as CIED combined with heart failure, coronary heart disease, and arrhythmia, were not considered. Second, the causes of patients’ fear of exercise and the mechanism of stress perception were not discussed in detail.Third,the included literatures were limited to studies published in Chinese and English. Fourth, only a few RCTs were included in this review,future studies should conduct or include high-quality primary studies.

## 6 Conclusion

This study integrated relevant evidence on sports risk stratification management, including exercise risk assessment, exercise prescription implementation, and the prevention and management of risk events. It also clarifies the role of nurses in evaluating, monitoring, and educating patients receiving cardiac rehabilitation. Our findings provides a basis for the formulation of clinically feasible rehabilitation programs. The management of symptoms of comorbidities (heart failure, arrhythmia, etc.) in patients with CIED should be strengthened. This can be achieved through nurse-led observation and risk assessment, nurses’ psychological intervention in patients with CIEDs, and health education for patients. Future studies should examine nursing interventions for the stress perception of frailty in patients after CIED.

## Data Availability

The original contributions presented in the study are included in the article/Supplementary Material, further inquiries can be directed to the corresponding author.
